# What primary care physician teachers need to sustain community based education in Japan

**DOI:** 10.1186/1447-056X-13-6

**Published:** 2014-04-28

**Authors:** Manabu Murakami, Hidenobu Kawabata, Masaji Maezawa

**Affiliations:** 1 International Relations Office, Graduate School of Medicine, Hokkaido University, North 15, West 7, 060-8638, Kita-ku, Sapporo, Japan; 2 Medical Education Development Center, Hokkaido University, Sapporo, Japan; 3 Hokkaido University, Sapporo, Japan

**Keywords:** Community based education, Semi-structured interview, Workshop, Physician needs, Non-monetary support

## Abstract

**Background:**

Community based education (CBE), defined as “a means of achieving educational relevance to community needs and, consequently, of implementing a community oriented educational program,” is reported to be useful for producing rural physicians in Western countries. However, why some physicians withdraw from their teaching roles is not well known, especially in Asian countries. The aim of this study was to clarify the requisites and obstacles for taking part in CBE.

**Methods:**

We combined two steps: preliminary semi-structured interviews followed by workshop discussions. First of all, we interviewed four designated physicians (all male, mean age 48 years) working in one rural area of Japan, with less than 10,000 residents. Secondly, we held a workshop at the academic conference of the Japan Primary Care Association. Fourteen participants attending the workshop (seven male physicians, mean age 45 years, and seven medical students (one female and six male), mean age 24 years) were divided into two groups and their opinions were summarized.

**Results:**

In the first stage, we extracted three common needs from interviewees; 1. Sustained significant human relationships; 2. Intrinsic motivation; and 3. Tangible rewards. In the second stage, we summarized three major problems from three different standpoints; A. Preceptors’ issues: more educational knowledge or skills, B. Learner issues: role models in rural areas, and C. System issues: supportive educational system for raising rural physicians.

**Conclusions:**

Our research findings revealed that community physicians require non-monetary support or intrinsic motivation for their CBE activities, which is in accordance with previous Western studies. In addition, we found that system support, as well as personal support, is required. Complementary questionnaire surveys in other Asian countries will be needed to validate our results.

## Background

For more than 20 years, members of a WHO study group have advocated the importance of community based education (CBE) [1,2]. According to this group, CBE is defined as “a means of achieving educational relevance to community needs and, consequently, of implementing a community oriented educational program [1]”. Some studies have shown that CBE is effective, from the standpoint of: the effect on recruitment of new community primary care physicians [3-5]; promoting professional development in students when they are exposed to positive role models [3]; and nurturing student motivation by giving them the opportunity to interact with patients or physicians on a practical level [3,6]. Not only students, but individual patients, have personal gain by participating in CBE, since it contributes to a feeling of “improved knowledge (about their disease)” or “enhanced self-esteem (as being part of student education in their community)” [7].

Community preceptors, including general physicians working in rural areas, seem to have an interest in teaching students and feel strong intrinsic motivation for their teaching role [8,9]. However, some obstacles such as too much clinical work and shortage of administrative support or financial compensation, may result in burnout and inability to sustain their educational activities [1,10,11]. Many studies in Western countries are trying to clarifying the factors contributing to the recruitment and retention of physicians who are willing to cooperate in community-based education [3,12,13]. Some successful examples of the factors have been reported in previous studies [14,15]. For example, in Australia, the Commonwealth government promoted a policy encouraging the enrolment of students who had a rural background [14]. In the U.S., Jefferson Medical College established a physician shortage area program (i.e. special program for admission and education to increase rural family physicians) [15]. These research results share some important similarities among admission policies, like accepting applicants who grew up in rural communities. Educational policies, like teaching in rural or small village locations, also seem to be important factors.

While teaching medical students in community ambulatory settings is a recent global trend [3,10,16], to our knowledge, there are few research reports about CBE in Asian countries. Particularly, in Japan, too much specialization of university departments has developed, and general clinical education is insufficient [17]. Furthermore, a severe shortage of general physicians working in rural communities has been reported over the past two decades [18]. Therefore, to promote CBE and increase the number of physicians working in rural areas, we explored requisites for the retention of physicians engages in CBE activities.

## Methods

We used a two-step “methodological triangulation [19]”, which involves two or more data collection methods to increase the validity of the results.

Focusing on their needs in the first stage, as a preliminary study, we interviewed four physicians who were actively engaging clinical and educational activities. An initial interviewee was designated by the chief director of the Japan Primary Care Association (JPCA) as experienced physicians. To decide following interviewees, we used snowball sampling [20], which is used when authors do not know how to reach potential participants, and participants recruit other participants from among their own acquaintances.

**Figure 1:**
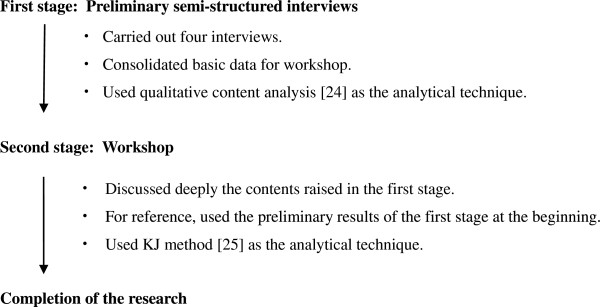
**Research process.** We used two steps: pilot interviews (the first stage) and the following workshop (the second stage) as the methodological triangulation [19].

In the second stage, we held a workshop at the academic conference of the JPCA in 2011, and summarized participants’ (including both physicians as representative preceptors and medical students as potential learners) overall opinions. Concerning the workshop, we sought out participants from among the members of the JPCA, and all candidates could apply freely without any restrictions. At first, we presented the results of the preliminary interviews to the participants, so that they could deepen their understandings about the research question. Secondly, group discussion about the problem took place. For more details about our research process, see Figure 1.

### First stage: preliminary semi-structured interviews

Participants were four male physicians (mean age 48 years), living in one northern area of Japan, who had been working in an area with less than 10,000 inhabitants for more than five years [21]. A qualitative approach with semi-structured interviews [22,23] was adopted in this study. Major interview topics, amended from previous studies [11,21] included: *1. What are the obstacles of rural physicians’ CBE/and possible solutions; 2. What motivates rural physicians to engage in their teaching roles and 3. What do rural physicians need to sustain their teaching activities?* (For more details, see “Interview guide for semi-structured interviews” section). All interviews lasted approximately 60 minutes and were transformed into verbatim script form. As the analytical method, we used qualitative content analysis [24] which Graneheim and Lundman introduced in nursing research. In brief, the scripts were independently analyzed by two researchers [MMu, HK] and repeated themes, which linked the latent meanings together in the categories (i.e. groups of content that shared a commonality but were mutually exclusive), identified. These two observers did cross-checking of the analyses. Finally, the senior author [MMa] supervised all analyses in the form of peer debriefing [20], and all authors approved the revised results.

### Interview guide for semi-structured interviews

(1) Opening (as introduction): Please introduce yourself to us. Please tell us about your working/teaching environment. (2) Main subject (in no fixed order from A. to C.): A. Obstacles: What are the obstacles of rural physicians’ community based education? What do you think are possible solutions? B. Motivational factors: What motivates rural physicians to engage in their teaching roles? What do you think about enhancing them? C. Needs: What do rural physicians need to sustain their teaching activities? What do you think about promoting their activities? (3) Closing (as supplement): Do you have any comments you want to add? Do you have anything else you forgot to say?

### Second stage: workshop

Seven male physicians (mean age 45 years) and seven medical students (one female and six males; mean age 24 years), were divided into two groups and participated in the workshop.

The attendee’s opinions were summarized using a useful and creative brainstorming analytical technique, called the “KJ method [25]”: a label-based manual method, well known and regularly taught in the field of business or administrative study in Japan. Details of the analytical method are available elsewhere [25], but in brief, the KJ method consists of four steps. 1) Making labels, on which participants’ ideas are written, using post-its. 2) Grouping labels of similar meanings together, and giving that group a title. 3) Arranging the titles on a white board and drawing a chart to represent the relationship of each title to the others. 4) Explaining the chart verbally. The results of grouped titles and the verbal explanations are presented in this report.

## Results

In the first stage with semi-structured interviews, we extracted three common needs from interviewees.

### Need 1. Sustained significant human relationships

All physicians insisted that sustained significant human relationships (i.e. forming a network between students and university faculties, as well as developing partnerships with other medical professions to work together) would potentially lead to an increase in the production of more rural physicians.

Participants expressed a strong desire to be able to form a network between students and university faculty, such as, “*I want to have the chance to be able to communicate with university faculty*.” In addition, some rural physicians pointed out the reason for having lost their educational motivation, when they got no response from their students, such as, “*I do not want to be responsible for students, because, they seem to lack enthusiasm…*”

### Need 2. Intrinsic motivation

All physicians suggested that, if they were to continue to engage in educational activities, they needed to foster a feeling of enjoyment or satisfaction in mentoring younger generations. The suggested that it may contribute to increasing their motivation to achieve ideal medical practice.

For example, one participant stated enjoyment in mentoring the younger generations with expectations as, “*In the hope of seeing future younger physicians, even one or two, become rural physicians someday. That’s why I must continue to work at the forefront*.” Another physician said, “*One day, I thought a lot about my reasons for living. (To be a good doctor is to raise a competent successor.) That’s one of reasons why I chose to be a rural physician*”.

### Need 3. Tangible reward

One physician suggested financial compensation or infrastructural subsidies. He commented about remunerative or material rewards by saying, “*Most of our time and energy goes toward mentoring students. It is natural we should be paid in proportion to the amount of work we do*”.

However, others stated that they primarily desired intrinsic matters rather than financial aid. For example, “*I want to get the chance to communicate with university faculty who administer the program. Mutual feedback is necessary,*” “*Regarding fostering younger doctors, inhabitants of rural communities are kind and calm. Many students are like rough but potential diamonds”.*

Based on these results, we discussed the research question further in the second stage of the study. Participants in the workshop presented their opinions clearly, and brought out three major problems to be solved from three different standpoints (i.e. the standpoint of preceptors, learners, and the system itself).

### Problem A. Preceptor issues: educational knowledge or skills

Some participating physicians discussed their need for further educational knowledge or skills as a major potential problem. One participant explained his interpretation about one of the reasons for this (Needs 1: Sustained significant human relationship) as the need for connection to educational specialists. “*If we were given the chance to go somewhere and meet educational specialists, we could learn about the technique of mentoring*”.

### Problem B. Learner issues: role models in rural areas

Both two groups presented that the problem of role models in rural areas. Some of the participants (especially students) cited (Need 2: Intrinsic motivation), and added that even if they hoped to raise younger physicians, there were not enough precedents. One representative student presenter pointed out the following in his presentation, “*Students do not have the opportunity to get to know family physicians or general physicians in their first or second year. So, they do not know that family physicians exist in rural areas*”.

### Problem C. System issues: a supportive educational system for producing rural physicians

Some participants discussed the lack of a supportive system, especially for producing rural physicians. They seemed to point out the effect of the organizational structure or culture in Japanese Universities with regards to the structure of educational activities as well as individual personal problems of physicians. For example, “*Japanese Universities do not traditionally have the know-how to produce rural physicians. Educators are not regarded highly in Japanese Universities*”.

(Need 3: Tangible rewards) did not lead to any major discussion in the workshop.

## Discussion

Four interviewees’ and 14 workshop participants desired non-monetary support or intrinsic motivation rather than tangible rewards (i.e. fiscal compensation or infrastructural subsidies). Our results were in accordance with similar studies of family physicians in Western countries [4,8,11,13] and suggest that financial compensation was the lowest ranking incentive among community physicians. Some studies that investigated preceptor identities have reported that community physicians enjoyed their educational activities, and were greatly satisfies by the fact that they were valued role models, and could promote their knowledge and skills among the next generation [8-10,12].

If our research results are valid, then we should promote CBE in many Asian countries. It may help resolve the issue of a lack of rural physicians in Asian countries, even when acknowledging the deficit of their loss in clinical productivity [1,10,11]. Geographical mal-distribution of doctors is especially evident and persistent in Japan [18,21], due to the medical training system, i.e. too much specialization in urban tertiary hospitals that have been in charge of training younger physicians [17,21]. Since increasing the number of community physicians’ “role models (Workshop Problem B)” is needed, it might be a useful strategy for recruiting younger rural physicians if CBE were promoted [3-5]. Our research results also suggest that it might be possible to reduce educational costs, owing to community preceptors’ ideas about professional responsibility [9,10] as well as community residents’ altruism [7]. In Japan, the National University Corporation Law was legislated in 2004 and each university takes responsibility for its own finances. This is one of the reasons why educational activities are perceived to have low priority in Universities’ achievements compared with research or clinical achievements. [17,26]. It might lead to the result that they felt they need to gain “more educational knowledge or skills (Workshop Problem A),” after they going to rural areas and engaging in educational activities. However, we might be able to solve this problem by investing the money saved from the previously mentioned non-necessity of educational costs, and establishing a “more supportive educational system (Workshop Problem C)”.

A limitation to this study is that it was carried out in one region of Japan only. Many primary care physicians in Asian countries, including Japan, come from various academic backgrounds and working environments. Therefore, the results of our study may not represent the overall characteristics of primary care physicians from all Asian countries. While our results are similar to prior studies carried out in Western countries, the strength of the evidence is still weak and we, therefore, have to examine the validity of this survey in other countries. We are planning to carry out such studies using quantitative questionnaire surveys to validate our results in the future.

In addition, this survey was carried out in an area where very strong male bias existed, therefore, attention should be paid when we interpret the results. We also have to clarify female doctors’ perceptions in future studies. A review from the UK [27], reporting an increase in the proportion of women doctors in general practice, revealed some interesting results such as women doctors look for personal support like role models or mentors, and system support like flexible training. We believe similar results would have been found in our study if we had interviewed women doctors and students.

Furthermore, participants in the workshop were more likely to be those who were active in the field of and had positive opinions about CBE. It would, therefore, be interesting to also survey those physicians and students who had negative images about CBE in further studies.

Fortunately, the admission policy by the Japanese government of selecting potential students who grew up in rural areas may lead to an increased number of rural physicians in the near future [28]. One example of reasonable success is Jichi Medical School (JMS), which was established in 1972 by government initiatives in Japan [28,29]. This school recruits two or three students from high schools in each prefecture, and their tuition fee is free during their 6 year undergraduate medical education period. The program of their system requires that the students spend some weeks in their home prefectures. JMS also prepares students for elective internship programs in rural areas. After graduation, the students have to spend 9 years in rural areas of their home prefecture. (i.e. 2 years postgraduate training and 7 years obligatory task.) If they refuse to fulfill the 9 years obligation, they have to pay for the all their university expenses, including tuition fees, with interest.

In addition to government initiatives, leadership among academic communities is also important for supporting rural physicians’ educational activities. The establishment of effective faculty development programs for community preceptors in medical schools or teaching medical centers is necessary. One such example, the Mountain Area Health Education Center in North Carolina [30], revealed that busy community preceptors needed highly concise materials prepared from supportive educational systems, and they hoped for close human relationships such as contact with university faculty, which was consistent with our research results.

The JPCA, the largest academic association of Japanese general practitioners, was established by the revolutionary integration of three Japanese primary care societies in 2010 [26]. It allows many Japanese primary care preceptors in rural areas to share information about useful educational programs for training younger physicians, which should provide a solid foundation for future CBE in Japan.

## Conclusions

Using two steps, a semi-structured interview, followed by a workshop, we attempted to clarify what rural physicians require to continue their participation in CBE, and the major problems remaining from the standpoint of students, preceptors, and the system itself. Our findings revealed that non-monetary support or intrinsic motivation seemed to be key points for the promotion of CBE. Furthermore, a system that provides not only personal, but educational support is also necessary. Further study in other Asian countries must be carried out to support our results.

### Ethical approval

Formal ethical approval was obtained from the Ethics Committee of Hokkaido University Graduate School of Medicine.

## Competing interests

This survey was partially supported by the Pfizer Health Research Foundation.

## Authors’ contributions

MMu designed the study. In the first stage, MMu and HK conducted interviews, and analysed preliminary data. MMa, the senior researcher, supervised all analyses, and all three authors discussed, revised, and approved the final result. In the second stage, all three authors cooperated closely in the planning of the workshop. MMu is the guarantor of this manuscript. All authors read and approved the final manuscript.
